# Machine Learning for Detecting Parkinson’s Disease by Resting-State Functional Magnetic Resonance Imaging: A Multicenter Radiomics Analysis

**DOI:** 10.3389/fnagi.2022.806828

**Published:** 2022-03-03

**Authors:** Dafa Shi, Haoran Zhang, Guangsong Wang, Siyuan Wang, Xiang Yao, Yanfei Li, Qiu Guo, Shuang Zheng, Ke Ren

**Affiliations:** ^1^Department of Radiology, Xiang’an Hospital of Xiamen University, School of Medicine, Xiamen University, Xiamen, China; ^2^School of Medicine, Xiamen University, Xiamen, China; ^3^Xiamen Key Laboratory for Endocrine-Related Cancer Precision Medicine, Xiang’an Hospital of Xiamen University, School of Medicine, Xiamen University, Xiamen, China

**Keywords:** Parkinson’s disease, amplitude of low-frequency fluctuation, radiomics, support vector machine, machine learning, biomarker, sensorimotor network

## Abstract

Parkinson’s disease (PD) is one of the most common progressive degenerative diseases, and its diagnosis is challenging on clinical grounds. Clinically, effective and quantifiable biomarkers to detect PD are urgently needed. In our study, we analyzed data from two centers, the primary set was used to train the model, and the independent external validation set was used to validate our model. We applied amplitude of low-frequency fluctuation (ALFF)-based radiomics method to extract radiomics features (including first- and high-order features). Subsequently, *t*-test and least absolute shrinkage and selection operator (LASSO) were harnessed for feature selection and data dimensionality reduction, and grid search method and nested 10-fold cross-validation were applied to determine the optimal hyper-parameter λ of LASSO and evaluate the performance of the model, in which a support vector machine was used to construct the classification model to classify patients with PD and healthy controls (HCs). We found that our model achieved good performance [accuracy = 81.45% and area under the curve (AUC) = 0.850] in the primary set and good generalization in the external validation set (accuracy = 67.44% and AUC = 0.667). Most of the discriminative features were high-order radiomics features, and the identified brain regions were mainly located in the sensorimotor network and lateral parietal cortex. Our study indicated that our proposed method can effectively classify patients with PD and HCs, ALFF-based radiomics features that might be potential biomarkers of PD, and provided further support for the pathological mechanism of PD, that is, PD may be related to abnormal brain activity in the sensorimotor network and lateral parietal cortex.

## Introduction

Parkinson’s disease (PD) is the second most common progressive neurodegenerative disease, affecting 1% of the population over 60 years (Lin et al., 2020; Ren et al., 2021), and it is becoming more and more prevalent and associated with increased mortality (Shu et al., 2021). The clinical symptoms of PD are heterogeneous, presenting a variety of motor symptoms (e.g., static tremor, bradykinesia, or rigidity) and non-motor symptoms (e.g., sensory and autonomic dysfunction, cognitive deficits, or disorders of mood) (Kim et al., 2017; Amoroso et al., 2018; Lin et al., 2020; Sheng et al., 2021). The diagnosis of PD is mainly based on clinical manifestations, imaging scans, and related biochemical examinations, which remain clinically challenging (Badea et al., 2017; Heim et al., 2017). However, accurate diagnosis of PD is essential for effective treatment and favorable prognosis. Moreover, even the main neural and pathophysiological mechanisms of PD are the degeneration of the nigrostriatal dopaminergic system; it cannot fully explain the heterogeneity of symptoms (Tuovinen et al., 2018; Sheng et al., 2021). The exact mechanism of PD is still not well understood (Tuovinen et al., 2018; Cao et al., 2020; Lin et al., 2020; Sheng et al., 2021). Therefore, quantifiable biomarkers are urgently needed for a more comprehensive understanding of the physiological mechanism of PD and improving the diagnosis accuracy.

Resting-state functional magnetic resonance imaging (rs-fMRI), as one of the most commonly used non-invasive techniques in neuroimaging, has been widely used in the diagnosis (Heim et al., 2017; Rubbert et al., 2019; Pang et al., 2021; Shi et al., 2021a), monitoring of treatment effects (Morgan et al., 2017; Ge et al., 2020), clinical score prediction (Hou et al., 2016), and conversion prediction (Hojjati et al., 2018) in neuropsychiatric diseases. The amplitude of low-frequency fluctuations (ALFF) is one of the most commonly used measurements of rs-fMRI. It can detect the amplitude of spontaneous, low-frequency oscillations of blood oxygen level-dependent signals to reflect the regularity and physiological state of neuron autonomous activity in different brain regions (Qian et al., 2020). This approach provides a reliable and sensitive measurement to characterize the spontaneous neural activity and has been widely used in PD (Cao et al., 2020; Tian et al., 2020; Pang et al., 2021; Shi et al., 2021b).

Radiomics is a data mining method proposed by Lambin et al. (2012), which can extract high-throughput features from medical images to characterize the characteristics of the lesions (Lambin et al., 2012; Aerts et al., 2014). Subsequently, the machine learning methods are performed for data mining. Recently, rs-fMRI-based radiomics has been applied to explore neurological disease biomarkers for disease diagnosis and underlying mechanisms (Sun et al., 2018; Mo et al., 2019; Wang Y. et al., 2020; Zhao et al., 2020), including PD (Cao et al., 2020; Shi et al., 2021b). However, the sample sizes of the above studies are limited and come from a single center, and the extracted features are the intensity histogram-based features.

In this study, we aimed to use data from two centers (one for model training and the other one for external validation of the model), and ALFF-based multi-order radiomics (including first- and high-order features) to identify potential neuroimaging biomarkers for distinguishing patients with PD from healthy controls (HCs) and explore the underlying mechanisms of PD. To the best of our knowledge, our study is the first to apply multi-order radiomics to identify PD biomarkers.

## Materials and Methods

### Participants

The data for this study were obtained from two independent public available databases. The primary set included 59 patients with PD and 41 age- and sex-matched HCs (Hu et al., 2015).^1^ The independent external validation set included 27 patients with PD and 16 HCs from the NEUROCON dataset, which were available at Functional Connectomes Project/International Neuroimaging Data-Sharing Initiative (FCP/INDI) (Badea et al., 2017).^2^ Clinical measurements were obtained, which included the Mini-Mental State Examination (MMSE) and the 17-item Hamilton Depression Rating Scale (HDRS-17) for the primary set and the Hoehn and Yahr staging scale (H&Y) and Unified Parkinson’s Disease Rating Scale (UPDRS, on/off medication) motor score for the external validation set. Demographic and clinical information of participants are listed in Table 1. Ethical approval was obtained by each institution, and all participants provided written informed consent.

**TABLE 1:** Demographic and clinical data of the two datasets.

	Primary set			External validation set		
	PD	HC	*P*-value	PD	HC	*P*-value
Age (years)^a^	56.46 ± 9.16 (32–71)	56.37 ± 5.01 (47–70)	0.95	68.70 ± 10.55 (45–86)	67.62 ± 11.89 (46–82)	0.76
Sex (M/F)^b^	35/24	20/21	0.32	16/11	5/11	0.12
Education (years)	11.31 ± 3.43 (2–19)	11.29 ± 4.58 (2–22)	0.99	−	−	−
MMSE^c^	29 (28–30) (24–30)	30 (29–30) (24–30)	0.017	−	−	−
HDRS-17^c^	9 (5–17) (0–28)	2 (1–3) (0–10)	<0.001			
H&Y	−	−	−	2 (2–2) (1.0–2.5)		−
UPDRS motor score (off)	−	−	−	28.33 ± 9.27 (10–43)	−	−
UPDRS motor score (on)	−	−	−	9.22 ± 5.27 (0–19)	−	−

*Data are presented as the mean ± SD (range) for normally distributed data or median (interquartile range) (range) for non-normally distributed data.*

*^a^The P-value was calculated using t-test.*

*^b^The P-value was calculated using the chi-square test.*

*^c^The P-value was calculated using the Mann-Whitney test. Abbreviations: MMSE, Mini-mental State Examination; HDRS-17, 17-item Hamilton Depression Rating Scale; H&Y, Hoehn and Yahr staging scale; UPDRS, Unified Parkinson’s Disease Rating Scale; M, male; F, female.*

### Data Acquisition

#### Primary Set

All subjects underwent structural and functional MRI scanning on a 3-T Siemens Verio scanner. Data acquisition parameters can be found in previous studies (Hu et al., 2015; Shi et al., 2021b). The structural images were acquired with high-resolution three-dimensional T1-weighted sequences [slices = 128, repetition time (TR)/echo time (TE) = 2,530/3.43 ms, field of view (FOV) = 256 × 256 mm, slice thickness/gap = 1.33/0.5 mm, matrix = 256 × 192, voxel size = 1 × 1.33 × 1.83 mm^3^, and flip angle (FA) = 7]. Rs-fMRI images were acquired with a gradient-recalled echo-planar imaging (GRE-EPI) pulse sequences (140 volumes, slices = 31, TR/TE = 2,000/30 ms, FOV = 220 × 220 mm, slice thickness/gap = 3.5/0.6 mm, matrix = 64 × 64, voxel size = 3.4 × 3.4 × 4.1 mm^3^, and FA = 90°).

#### External Validation Set

All subjects underwent structural and functional MRI scanning on a 1.5-T Siemens Avanto scanner. Data acquisition parameters can be found in the previous study (Badea et al., 2017) and online (see text footnote 2). The structural images were acquired with T1-weighted magnetization prepared rapid acquisition gradient-echo sequences (TR/TE = 1,940/3.08 ms and voxel size = 0.97 × 0.97 × 1 mm^3^). Rs-fMRI images were acquired with EPI sequences (137 volumes, slices = 27, TR/TE = 3,480/50 ms, voxel size = 3.8 × 3.8 × 5 mm^3^, and FA = 90°).

### Data Preprocessing and Amplitude of Low-Frequency Fluctuation Calculation

In this study, the data preprocessing was performed using the toolbox for Data Processing and Analysis of Brain Imaging (DPABI) (Yan et al., 2016).^3^ The primary set has completed the data preprocessing and ALFF calculation, and the processing flow is detailed in the previous study (Hu et al., 2015). A similar procedure as described above was used for processing the external validation set data. In brief, the preprocessing procedures included the following: removal of the first six time points (20.88 s); slice timing and spatial realignment (subjects with head motion >2.5 mm or >2.5° were excluded); segmentation of 3D T1-weighted anatomical images by new segment and registration by the Diffeomorphic Anatomical Registration Through Exponentiated Lie Algebra (DARTEL); spatial normalization by DARTEL and resampling (3 × 3 × 3 mm^3^); smooth with a 6-mm full-width-half-maximum Gaussian kernel; band-pass filter (0.01–0.10 Hz); linear drift, nuisance signal (white matter, cerebrospinal fluid, and global signal), and 24 head motion parameters were removed. Subsequently, we obtained the mean ALFF maps by DPABI’s default algorithm.

### Feature Extraction

**FIGURE 1:**
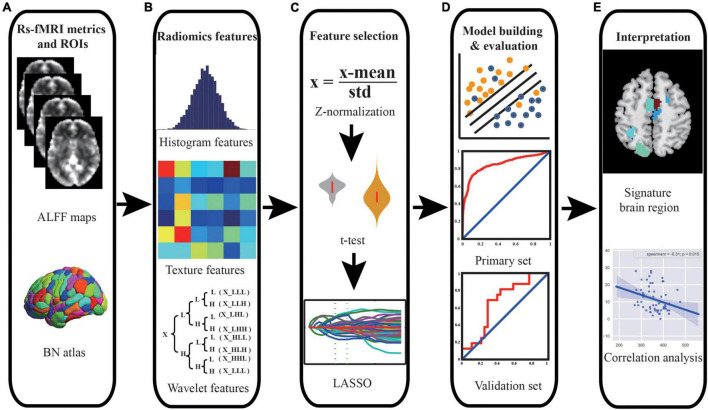
Schematic outline of the whole study analysis procedure. **(A)** ALFF maps and brain segmentation with Brainnetome 246 atlas. **(B)** Intensity histogram-, texture-, and wavelet transformation-based features were extracted from ALFF images. **(C)** Feature selection was performed using *t*-test and LASSO to select significant features and reduce dimensionality. **(D)** SVM model was constructed, and ROC curve analysis was employed to quantify the performance of the classifier in the primary set and independent external validation set. **(E)** The discriminative features were identified, and correlation analyses were performed to explain the underlying pathological mechanism of PD. Abbreviations: ALFF, amplitude of low-frequency fluctuation; BN, Brainnetome; LASSO, least absolute shrinkage and selection operator; SVM, support vector machine; ROC, receiver operating characteristic; PD, Parkinson’s disease.

The mean ALFF maps were segmented into 246 regions of interest (ROIs) using the Brainnetome 246 atlas (Supplementary Material). In this study, a total of 432 multi-order radiomics features were extracted from each ROI, including first-order intensity histogram-based features (15 features), texture features (33 features), and features of wavelet transformation in eight directions [(15 + 33) × 8 = 384 features]. In our study, the intensity histogram-based features are first-order features, which are used to characterize the gray level intensity in the image, using first-order statistics, calculated from the histogram of all voxels in the image. The texture features and wavelet features are high-order features. The texture features were able to quantify the spatial heterogeneity of the intensity level in the image. For wavelet features, wavelet filters are applied to the original images to convert original images to versions that focus on the information at different scales. Wavelet decomposition with all possible combinations of high (H)- or low (L)-pass filters in each of the three dimensions (LLL, LLH, LHL, LHH, HLL, HLH, HHL, and HHH) is applied. In this study, the first-order and texture features of eight directions were calculated. The definitions and detailed descriptions of the features can also be found in previous studies (Aerts et al., 2014; Feng et al., 2018; Zhao et al., 2020; Cui et al., 2021; Peng et al., 2021) and are listed in the Supplementary Material. The whole feature extraction process is illustrated in Figures 1A,B.

### Feature Selection, Model Construction, and Evaluation

**FIGURE 2:**
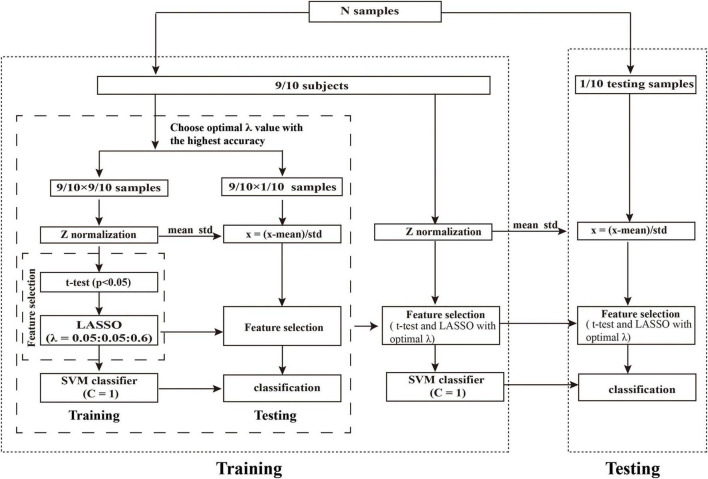
Schematic overview of the nested 10-fold cross-validation classification framework. We determined the optimal λ by grid-search from the set of (0.05, 0.10,…,0.60) with 10-fold cross-validation. The λ with the highest classification accuracy was selected as the optimal λ. Abbreviations: LASSO, least absolute shrinkage and selection operator; SVM, support vector machine.

In our study, we used the primary set for hyper-parameter optimization, feature selection, and model training and used the independent external validation set for external validation of the model. For feature selection, *t*-test and least absolute shrinkage and selection operator (LASSO) were applied, and the support vector machine (SVM) model with a linear kernel and default parameter value (i.e., *C* = 1) was chose as the classifier. The performance of the model was evaluated with receiver operating characteristic (ROC) curve analysis. In addition, the independent external dataset was applied for validating the generalization of our model. The whole procedure is illustrated in Figures 1C,D, 2.

First, we performed *Z*-score standardization on the features to reduce the influence of the different units imposed by the units of each feature and improve the performance of the model. The normalization of the primary and validation set were performed, respectively. Subsequently, we applied the *t*-test (*P* < 0.05) to select the features with significant differences between the patients with PD and HCs. Then, LASSO logistic regression was utilized to further reduce the dimensionality of the data. For LASSO logistic regression, the regularization parameter λ controls the number of model features and affects the performance of the model. So, the grid search method was optimized to determine the optimal hyper-parameter λ. According to the previous study (Chen et al., 2017; Zhao et al., 2018), the value of λ in our study was set to (0.05, 0.10, …, 0.60). The nested 10-fold cross-validation method (Ding et al., 2015, 2017; Zhao et al., 2018; Wottschel et al., 2019; Tu et al., 2020; Zhou B. et al., 2020) was performed to determine the optimal hyper-parameter λ of LASSO and evaluate the performance of the model. The outer 10-fold cross-validation was applied to estimate the performance of the model, and the inner 10-fold cross-validation was performed to determine the optimal hyper-parameter (optimal λ), in which the λ with the highest accuracy was selected as the optimal λ value.

To avoid the category information leakage, *t*-test and LASSO were carried out in a training set of inner 10-fold cross-validation, not for all subjects. Specifically, in each fold of the inner 10-fold cross-validation procedure, we had conducted the above *t*-test and LASSO on all subjects except one fold that was taken out. In other words, *t*-test and LASSO were only performed in the training set in the inner training set; no statistical tests were performed on the independent hold-out test data (inner and outer test set). Thus, analyses were unbiased in the sense that the training features were selected independently of test subjects. The whole procedure of nested 10-fold cross-validation was illustrated in Figure 2. To obtain unbiased estimates of classification error, we repeated the nested 10-fold cross-validation framework 20 times (Oh et al., 2019; Lin et al., 2020).

For model construction, we used an SVM to construct the model, where the SVM model adopted linear kernel function and default parameters (i.e., *C* = 1). The 10-fold cross-validation method (repeated 20 times) was applied to evaluate the performance of the SVM model. The mean accuracy, area under the curve (AUC), sensitivity, specificity, precision, F1 score, and balance accuracy across all folds (10-folds) and all repetitions (20 times) (Chen et al., 2016; Chen X. et al., 2017; Zhao et al., 2018) were employed to quantify the performance of the classifier. The accuracy, sensitivity, specificity, precision, F1 score, and balance accuracy were defined as follows:

Accuracy=(TP+TN)/(TP+TN+FP+FN)

Sensitivity=TP/(TP+FN)

Specificity=TN/(TN+FP)

Precision=TP/(TP+FP)

Recall=TP/(TP+FN)

F1⁢score=2×Precision×Recall/(Precision+Recall)

Balance⁢Accuracy=0.5×(Sensitivity+Specificity)

where TP represents the number of positive samples correctly classified; TN represents the number of negative samples correctly classified; FP represents the number of negative samples incorrectly classified; FN represents the number of positive samples incorrectly classified.

To obtain the final model, all the participants in the primary set were used to train the model with the optimal λ value (Shen et al., 2019; Zhao et al., 2020). Due to the different data of each fold, the optimal hyper-parameter might be different. We chose the λ with the highest frequency selected in all folds as the optimal hyper-parameter. In addition, to evaluate the generalization of the model, the independent external validation set was conducted to validate the performance of our model, where the model parameters (linear kernel function, *C* = 1) and selected features were the same as our final model. The accuracy, AUC, sensitivity, specificity, precision, F1 score, and balance accuracy were calculated to quantify the performance of the classifier in the external validation set.

To test the significance of model performances (AUC and accuracy), permutation tests were performed (Tang et al., 2017; Shen et al., 2019; Tian et al., 2020). Specifically, we shuffled the class labels (PD or HC) 1,000 times without replacement and performed the above-mentioned feature selection and model construction analysis process each time to obtain the permutated accuracies and AUCs. The *P*-value was defined as follows:

P=(1+N)GP/(1+N)

where *N*_GP_ represents the number of permutations that obtained greater accuracy or AUC than the actual value, and *N* was the times of permutation. In this study, the value of *N* is 1,000. We performed this analysis on the primary and external validation set, respectively.

### Identification of Discriminative Features

Since we implemented 10-fold cross-validation to evaluate the performance of our model, the training sets were different in each fold, and the selected features were also different. We sorted the selected feature frequencies and selected features in the top 10 discriminative regions as discriminative features (Zhou B. et al., 2020; Figure 1E). In each fold, we could also obtain feature weights. We calculated the mean weight of discriminative features across all folds. The greater the absolute value of the feature weight, the greater the contribution to the model.

### Relationship Between the Discriminative Features and Clinical Measurements

Spearman’s correlation coefficients were calculated to assess the association between the discriminative features and clinical measurements of patients with PD in the primary and external validation set (Figure 1E). *P* < 0.05 was considered statistically significant.

## Results

### Demographic and Clinical Information

The demographic and clinical characteristics of the participants in the primary and external validation set are summarized in Table 1. There were no significant differences in age, sex, and education duration between patients with PD and HCs (*P* > 0.05). The MMSE and HDRS-17 of patients with PD were significantly lower/higher than that of HCs in the primary set (*Z* = −2.39, *P* = 0.017; *Z* = −7.07, *P* < 0.001, respectively).

### Classification Performance

**FIGURE 3:**
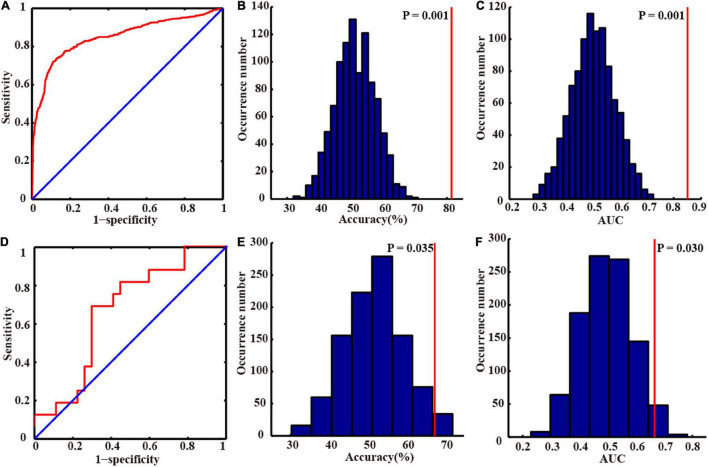
Classification performances in the primary and independent external validation sets. The receiver operating characteristic curves of the primary **(A)** and independent external validation sets **(D)**. The distributions of the permutated accuracy values of the primary **(B)** and validation set **(E)**. The distributions of the permutated AUC values of the primary **(C)** and validation set **(F)**. The red line indicates the values obtained using the real labels. Abbreviation: AUC, area under the curve.

In our study, we applied the grid search method to determine the optimal hyper-parameter λ of nested 10-fold cross-validation in the primary set. The mean accuracy was 81.45%, and AUC was 0.850 in the primary set. We chose the λ with the highest frequency selected in all folds as the optimal λ (λ = 0.45, Supplementary Figure 1) and constructed the final model. In the external validation set, our model also achieved great model generalization (accuracy = 67.44% and AUC = 0.667). The permutation test showed that the AUCs and accuracies were significantly higher than chance (*P* < 0.05). More detailed results are shown in Table 2 and Figure 3.

**TABLE 2:** Classifier performances in the primary and external validation sets.

	Accuracy	AUC	Sensitivity	Specificity	Precision	F1 score	Balance accuracy	*P*-value (accuracy)	*P*-value (AUC)
Primary set	81.45%	0.850	86.86%	73.66%	82.59%	83.68%	80.26%	0.001	0.001
Validation set	67.44%	0.667	70.37%	62.50%	76.00%	73.08%	66.44%	0.035	0.030

*Abbreviation: AUC, area under the curve.*

### Discriminative Features

**FIGURE 4:**
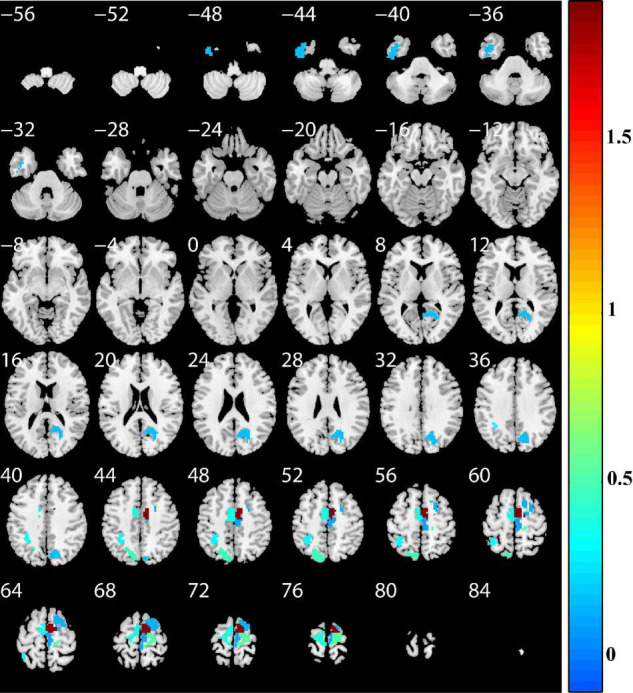
Discriminative brain regions. The discriminative regions included the bilateral superior frontal gyrus, precentral gyrus, right paracentral lobule, precuneus, left inferior temporal gyrus, and superior parietal lobule. The color bar value represents the absolute value of the weight value of the brain regions.

To determine which features contributed the most to the classification of patients with PD, we reported discriminative features and the feature weights. The features of the top 10 discriminative regions were selected as discriminative features in this study (Table 3 and Figure 4). The discriminative regions (including 17 features) included the bilateral superior frontal gyrus [SFG, SFG_R_7_4, and SFG_L(R)_7_5], precentral gyrus [PrG, PrG_L(R)_6_4], right paracentral lobule (PCL, PCL_R_2_2), precuneus (PCun, PCun_R_4_3), left inferior temporal gyrus (ITG, ITG_L_7_3), and superior parietal lobule (SPL, SPL_L_5_2, and SPL_L_5_3). The brain regions were mainly located in the frontal lobe, especially SFG.

**TABLE 3:** Discriminative features for patients with PD classification.

Lobe	Gyrus regions	Anatomical and modified cyto-architectonic descriptions	Feature	Weight
Frontal lobe	PrG_L_6_4	Area 4 (trunk region)	Minimum	0.2093
Frontal lobe	PrG_R_6_4	Area 4 (trunk region)	Minimum	−0.0612
Temporal lobe	ITG_L_7_3	Rostral area 20	Mean-HHL	−0.1392
Frontal lobe	SFG_L_7_5	Medial area 6	Median-HLL	−0.3554
Parietal lobe	SPL_L_5_3	Lateral area 5	Minimum-LLL	−0.3194
Frontal lobe	SFG_R_7_5	Medial area 6	Minimum-HLL	−0.3867
Frontal lobe	SFG_R_7_5	Medial area 6	Range-HLL	0.2307
Parietal lobe	PCun_R_4_3	Dorsomedial parietooccipital sulcus	Entropy-HHH	−0.1593
Frontal lobe	PrG_R_6_4	Area 4 (trunk region)	CT-HLL	0.3091
Frontal lobe	SFG_R_7_5	Medial area 6	Contrast-LHH	−0.4821
Frontal lobe	PrG_R_6_4	Area 4 (trunk region)	Correlation-HLL	0.1678
Parietal lobe	SPL_L_5_2	Caudal area 7	Homogenetity 2-HHH	0.4561
Frontal lobe	PCL_R_2_2	Area 4 (lower limb region)	IMC1-HHH	−0.0929
Frontal lobe	SFG_R_7_4	Dorsolateral area 6	SRE-HLH	−0.1312
Frontal lobe	SFG_R_7_5	Medial area 6	GLN-HHL	0.2744
Frontal lobe	PrG_L_6_4	Area 4 (trunk region)	GLN-HHH	0.1876
Frontal lobe	SFG_R_7_5	Medial area 6	RLN-HHH	−0.4933

*Abbreviations: PD, Parkinson’s disease; PrG, precentral gyrus; ITG, inferior temporal gyrus; SFG, superior frontal gyrus; SPL, superior parietal lobule; PCun, precuneus; SPL, superior parietal lobule; PCL, paracentral lobule; CT, cluster tendency; IMC, informational measure of correlation; SRE, short-run emphasis; GLN, gray level non-uniformity; RLN, run-length non-uniformity; L, left; R, right.*

### Correlations Between the Discriminative Features and Clinical Measurements

**FIGURE 5:**
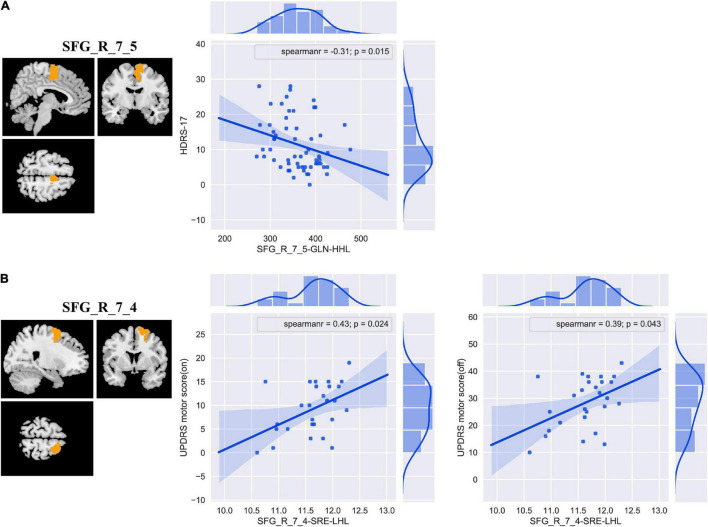
The correlation analyses between the discriminative features and clinical measurements in patients with PD in the primary **(A)** and external validation **(B)** set. Abbreviations: SFG, superior frontal gyrus; HDRS-17, 17-item Hamilton Depression Rating Scale; UPDRS, Unified Parkinson’s Disease Rating Scale; GLN, gray level non-uniformity; SRE, short-run emphasis; PD, Parkinson’s disease.

The results of correlation analyses are shown in Figure 5. In primary set, SFG_R_7_5-GLN-HHL was negatively correlated with HDRS-17 (Spearman’s correlation *r* = −0.31 and *P* = 0.015). In addition, in external validation set, we found positive correlations between SFG_R_7_4-SRE-LHL and UPDRS motor score (on medication) and UPDRS motor score (off medication) (Spearman’s correlation *r* = 0.43, *P* = 0.024; Spearman’s correlation *r* = 0.39, *P* = 0.043).

## Discussion

In our study, we selected brain region ROIs and extracted radiomics features based on Brainnetome 246 atlas, including intensity histogram-, texture-, and wavelet transformation-based features, and applied an SVM classifier to construct a model to classify patients with PD and HCs. We found that the classification accuracy of the model was 81.45%, and the AUC was 0.850 in the primary set. In the independent external validation set, our model has good generalization ability with an accuracy of 67.44% and an AUC of 0.667. More importantly, we are the first to apply multi-order (including first- and high-order features) radiomics to identify PD biomarkers, and our study demonstrated that radiomics features may be potential biomarkers of PD.

Previous studies have confirmed the value of rs-fMRI in neuropsychiatric diseases (Szewczyk-Krolikowski et al., 2014; Hu et al., 2015; O’Callaghan et al., 2016). Recently, with the development of machine learning technologies, more and more studies have used machine learning methods to explore the classification, prognosis prediction, and physiological mechanism of neuropsychiatric diseases, including PD (Cao et al., 2020; Lin et al., 2020; Pang et al., 2021; Shu et al., 2021; Talai et al., 2021; Zhang et al., 2021). The ROI-based feature extraction is the most commonly used feature extraction method (Wang L. et al., 2020; Zhao et al., 2020; Shi et al., 2021b; Talai et al., 2021), and it is a useful method to reduce the data dimensionality (Wang L. et al., 2020). Functionally defined parcelation and high spatial resolution segmentation might be able to detect a more significant difference, and the anatomical boundary might not match the functional boundary that has been reported in previous literature (Rosenberg et al., 2016; Chen et al., 2018). Therefore, we chose Brainnetome 246 atlas to segment brain region ROIs in our study. The previous ROI-based feature extraction methods mostly only extracted intensity histogram-based features (Peng et al., 2017; Cao et al., 2020; Jin et al., 2020; Tian et al., 2020; Zhou B. et al., 2020). In recent years, the value of high-order features (texture and wavelet features) had been confirmed and widely used in various studies (Feng et al., 2018; Mo et al., 2019; Zhao et al., 2020; Shu et al., 2021). To the best of our knowledge, the application of multi-order radiomics (including first- and high-order features) on PD has not been reported. We found that our method achieved perfect classification performance (accuracy = 81.45% and AUC = 0.850) and also obtained great performance in the independent external validation set (accuracy = 67.44% and AUC = 0.667), indicating that our model had good generalization (Zhao et al., 2020). In addition, our study indicates that the features that significantly contributed to the classification were mainly high-order features (wavelet features). Additionally, only two of the 17 discriminative features identified by this study were first-order features, the remaining 15 features were high-order features, and the brain region identified by both the two features based on first-order features was also identified by high-order features. Those results confirmed the value of high-order radiomics features, which may be a better characterization of lesions than first-order radiomics features and more suitable as potential biomarkers for PD (Feng et al., 2018; Mo et al., 2019; Zhao et al., 2020). Those findings are consistent with the previous results mentioned earlier.

Radiomics can extract high-throughput features from medical images (Lambin et al., 2012; Aerts et al., 2014; Feng et al., 2018; Sun et al., 2018; Zhao et al., 2020), and the dimension of features is much higher than the sample size, which may easily make the model fall into a “curse of dimension” and model overfitting. Especially, we extracted not only first-order features but also high-order features. In addition, many features may be uninformative, irrelevant, or redundant; therefore, feature selection and data dimensionality reduction were performed before our SVM model construction. First, we performed the *t*-test (*P* < 0.05) to identify the significant features between the patients with PD and HCs. Subsequently, LASSO logistic regression was performed to choose the most important features for classification. The *t*-test is a filter method to reduce the data dimensionality. It can simply and quickly remove features with no or less information and has been widely used in machine learning (Lanka et al., 2020a; Tu et al., 2020; Wang Y. et al., 2020). It is a built-in algorithm of many software, such as BrainNetClass (Zhou Z. et al., 2020), MALINI (Lanka et al., 2020b), and MANIA (Grotegerd et al., 2014), and it is recommended as the first step in data dimensionality reduction (Lanka et al., 2020b). LASSO is very suitable for high-dimensional data processing (Chen X. et al., 2017; Zhao et al., 2018; Wang Y. et al., 2020; Shu et al., 2021). It can select the most important features, compress unimportant feature coefficients to zero, and eliminate multicollinearity between features to achieve the purpose of data dimensionality reduction and feature selection (Chen et al., 2016; Chen X. et al., 2017; Zhao et al., 2018; Huang et al., 2020; Wang Y. et al., 2020; Shu et al., 2021). We used the grid search method (λ = 0.05, 0.10…0.60) and nested 10-fold cross-validation to determine the optimal lasso hyper-parameter λ and evaluate the performance of the model. The outer 10-fold cross-validation was applied to estimate the performance of the model, and the inner 10-fold cross-validation was performed to determine the optimal hyper-parameter (optimal λ). In our study, in each fold of 10-fold cross-validation, the mean number of remaining features after LASSO analysis was 16. LASSO analysis greatly reduced the number of features, and most of the features appeared repeatedly in multiple folds. Those confirmed the effectiveness of LASSO and the stability of the features that we identified (Feng et al., 2018; Mo et al., 2019; Zhao et al., 2020; Shu et al., 2021). Those are consistent with the above-mentioned previous results.

Support vector machine is one of the most commonly used machine models, especially in neuroimaging studies in which the sample size is relatively limited (Hong et al., 2017; Tian et al., 2020; Shu et al., 2021; Talai et al., 2021; Zhang et al., 2021). SVM incorporates several advantageous properties to reduce overfitting and deliver good generalization performance despite a small sample size (Hong et al., 2017; Mo et al., 2019). The SVM classifier was selected to construct the model in our study. The results demonstrated that our method achieved perfect classification performance and also obtained great generalization performance in the external validation set (Table 2 and Figure 3).

We found that, in addition, the discriminative regions included bilateral SFG, PrG, right PCL, precuneus, left ITG, and SPL. The features of bilateral SFG and PrG served as the most important features in classification, and the features of SFG were correlated with clinical measurements [HDRS-17 and UPDRS motor score (on/off medication)]. The SFG and PrG are important components of the sensorimotor network, which plays a central role in the preparation and execution of motor functions. Multiple previous studies have reported the sensorimotor network dysfunction in patients with PD (Tuovinen et al., 2018; Rubbert et al., 2019; Cao et al., 2020; Chen et al., 2021; De Micco et al., 2021; Wang et al., 2021). Abnormal brain activation of SFG and PrG was also revealed in previous studies (Lin et al., 2017; Peng et al., 2017; Cao et al., 2020; Guo et al., 2020; Tian et al., 2020; Pang et al., 2021). Our study found that the SFG features were correlated with UPDRS motor score and HDRS, indicating the association between SFG and PD symptoms. Many studies indicated that the lateral parietal cortex (including SPL) plays an important role in PD with movement dysfunction (Tian et al., 2020), and the precuneus is located in SPL and involved in visuospatial processing, episodic memory, self-reflection, and consciousness (Guo et al., 2020). Abnormal spontaneous brain activities in right PCL (Chen B. et al., 2017; Guo et al., 2020; Sheng et al., 2021; Suo et al., 2021), left ITG (Jiang et al., 2016; Chen B. et al., 2017; Guo et al., 2020; Tian et al., 2020), and STG (Chen B. et al., 2017; Lin et al., 2017) in patients with PD had also been reported. Those are consistent with the previous results. Our results indicated that our method could effectively identify the brain spontaneous abnormal activities of patients with PD and could be used as a potential biomarker for PD and provided further support for the pathological mechanism of PD, that is, PD may be related to abnormal brain activity in the sensorimotor network and lateral parietal cortex.

Several issues need to be addressed in this study. First, although the sample size of our study is relatively larger than that of some machine learning studies (Hou et al., 2016; Tang et al., 2017) and our data come from two centers, the sample size is still relatively limited. Therefore, future study with more participants and multiple centers will improve the generalizability of our findings. Second, although the field strength of the MRI scanners and data acquisition parameters of the two datasets are different, we analyzed the data of the two centers separately. One was used to train the model; the other one was used to validate the performance of the model. Both the two datasets had good classification performance, which further indicated the good classification performance and generalization of our model. Third, it has been reported that combining multimodal data and clinical data can improve the performance of the machine learning model (Shi et al., 2021a; Talai et al., 2021), but the primary set in this study only contained ALFF data. A subsequent study should incorporate other modal MRI data, metrics, and clinical data to construct and evaluate the model. Fourth, previous studies (Lin et al., 2017; Pang et al., 2021) have reported that patients with PD have structural and functional abnormalities in the cerebellum, but the Brainnetome 246 atlas we used in this study did not include the cerebellum.

## Conclusion

This study uses the ALFF-based radiomics method to extract multi-order features and uses an SVM to construct the model to classify patients with PD and HCs. Good model performances were achieved in both primary and independent external validation sets, most of the discriminative features were high-order features and moderately related to PD symptom scores, and the identified brain regions were mainly located in the sensorimotor network and lateral parietal cortex. Our results indicated that our proposed method can effectively classify patients with PD and HCs, in which ALFF-based radiomics features might be potential biomarkers of PD, and provided further support for the pathological mechanism of PD, that is, PD may be related to abnormal brain activity in the sensorimotor network, thalamus, and lateral parietal cortex.

## Data Availability Statement

Data used in this study were obtained from two independent publicly available databases: primary set downloaded from http://dx.doi.org/10.6084/m9.figshare.1433996 and external validation set downloaded from http://fcon_1000.projects.nitrc.org/indi/retro/parkinsons.html. Further inquiries can be directed to the corresponding author.

## Ethics Statement

The studies involving human participants were reviewed and approved by Medical Research Ethical Committee of Nanjing Brain Hospital and National Institute for Research and Development in Informatics, Bucharest, Romania. The patients/participants provided their written informed consent to participate in this study.

## Author Contributions

DS conducted the experiment, performed the data processing and analysis, and wrote and edited the manuscript. HZ and GW collected the data, performed the data processing and analysis, and edited the manuscript. SW, XY, YL, QG, and SZ collected the data and performed the data processing and analysis. KR supervised the whole study. All authors contributed to this study and approved the submitted version.

## Conflict of Interest

The authors declare that the research was conducted in the absence of any commercial or financial relationships that could be construed as a potential conflict of interest.

## Publisher’s Note

All claims expressed in this article are solely those of the authors and do not necessarily represent those of their affiliated organizations, or those of the publisher, the editors and the reviewers. Any product that may be evaluated in this article, or claim that may be made by its manufacturer, is not guaranteed or endorsed by the publisher.

## Footnotes

1
http://dx.doi.org/10.6084/m9.figshare.1433996

2
http://fcon_1000.projects.nitrc.org/indi/retro/parkinsons.html

3
http://rfmri.org/DPABI
